# Sleep hygiene and sleep quality among yoga and naturopathy medical students in India: a multisite cross-sectional study

**DOI:** 10.3389/frsle.2025.1459750

**Published:** 2025-04-09

**Authors:** Karishma Silwal, Prakash Babu Kodali, Hemanshu Sharma, Nair Dhiren Ajit, Abhay M. Shankaregowda, Renjish Mohanan, M. Jerin Subha, K. Vibhas, S. Sivaraman, Kinjal Bhalavat, V. R. Dhilip, Jyoti Nair, Vanitha Shetty, A. N. Vineetha, Dhanya U. M. Kumar, Rakesh Gupta, Ayswarya Rohini Pandian, Vakeel Khan, Pradeep M. K. Nair

**Affiliations:** ^1^Department of Naturopathy, Sant Hirdaram Medical College of Naturopathy and Yogic Sciences for Women, Bhopal, Madhya Pradesh, India; ^2^Department of Naturopathy, Central University of Kerala, Kasaragod, Kerala, India; ^3^Department of Naturopathy, Swami Vivekanand Subharti University, Meerut, Uttar Pradesh, India; ^4^Department of Naturopathy, GTN Medical College of Naturopathy and Yogic Science Research Centre, Dindigul, Tamil Nadu, India; ^5^Department of Naturopathy, Excel Medical College for Naturopathy and Yoga, Komarapalayam, India; ^6^Department of Naturopathy, Balvantrai Mehta Naturopathy Centre, Vadodara, India; ^7^Department of Naturopathy, JSS Institute of Naturopathy and Yogic Sciences, Coimbatore, India; ^8^Department of Naturopathy, Swamy Vivekanandha Naturopathy and Yoga Medical College, Salem, India; ^9^Department of Naturopathy, Alva's College of Naturopathy and Yogic Sciences, Moodbidri, Karnataka, India; ^10^Department of Naturopathy, University College of Naturopathy and Yogic Sciences, Jodhpur, India; ^11^Department of Naturopathy, International Institute of Yoga and Naturopathy Medical Sciences, Chengalpattu, India; ^12^Department of Integrative Oncology, Mirakle Integrated Health Centre, Pollachi, India

**Keywords:** sleep hygiene, sleep quality, naturopathy, yoga, poor sleep, Internet addiction

## Abstract

**Introduction:**

Poor sleep quality and sleep hygiene among medical students is a concern, with limited data on students from alternative medical systems.

**Methods:**

This cross-sectional study assessed 1,151 undergraduate yoga and naturopathy medical students from 10 Indian colleges. A structured questionnaire was used to collect sociodemographic and lifestyle data, while sleep quality, sleep hygiene, depression, anxiety, stress, and Internet addiction were evaluated using the Pittsburgh Sleep Quality Index (PSQI), the Sleep Hygiene Index (SHI), the Depression Anxiety Stress Scale (DASS-21), and the Internet Addiction Test (IAT). Logistic regression models were employed to compute adjusted odds ratios (AORs) as measures of association.

**Results:**

The average SHI and PSQI scores were 22.49 (±6.34) and 6.52 (±3.01), respectively. Poor sleep quality was reported by 59.8 and 24.7% had poor sleep hygiene. Severe depression (AOR = 5.15) and anxiety (AOR = 2.31) were linked to poor sleep hygiene, while severe stress (AOR = 0.55) was associated with poor sleep quality. Family residence was linked to lower odds of poor sleep hygiene (AOR = 0.14 for male participants, AOR = 0.26 for female participants) and better sleep quality (AOR = 1.46). Poor sleep hygiene was associated with sugary beverage consumption (AOR = 2.02), fried/packaged foods (AOR = 5.06 weekly, AOR = 8.52 daily), Internet addiction (AOR = 21.87 for male participants, AOR = 9.57 for female participants), and late device use (AOR = 3.80 for female participants).

**Discussion:**

Despite early exposure to lifestyle principles, yoga and naturopathy students experience poor sleep quality. Contributing factors include poor sleep hygiene, anxiety, stress, unhealthy eating habits, and Internet addiction. Targeted interventions are needed to improve sleep hygiene and overall wellbeing.

## Introduction

Sleep is a fundamental biological necessity crucial for cognitive functioning, mental wellbeing, and physical health (Ramar et al., 2021). Poor sleep quality and sleep deprivation are serious issues across the globe, affecting nearly one-third of the population (Grandner, 2019). While issues related to sleep and its quality are a concern among the general population, this is equally prevalent among students, with a prevalence rate ranging from 60 to 75% (Guo et al., 2023; Lankrew Ayalew et al., 2022; Lund et al., 2010). Medical students often face sleep deprivation due to academic demands, clinical responsibilities, and lifestyle factors (Azad et al., 2015). Addressing the factors that influence sleep quality is important, as inadequate sleep can be linked to various health issues, daytime sleepiness, and potential challenges with academic performance (Nsengimana et al., 2023).

*Sleep hygiene* refers to a set of behaviors and environmental modifications (such as engaging in regular exercise, minimizing phone usage, maintaining a consistent sleep schedule, refraining from stimulants before bedtime, and avoiding extended daytime naps) adopted to ensure good sleep quality (Chow, 2022). Maintaining optimal sleep hygiene is associated with sleep quality (Miller et al., 2022). However, studies suggest the prevalence of suboptimal sleep hygiene practices among medical students that serve as predictors for poor sleep quality (Molla and Wondie, 2021; Brick et al., 2010).

India's medical ecosystem is plural, where along with biomedicine, traditional medical systems, such as Ayurveda, Yoga and Naturopathy, Unani, Siddha, Homeopathy, and Sowa-Rigpa, are prominently practiced. As of 2019, India had more than 0.64 million doctors following traditional medical systems registered under the Ministry of Ayush (Ayurveda, Yoga and Naturopathy, Unani, Siddha, and Homeopathy) (National Health Profile (NHP) of India, 2021). There are more than 740 Ayush colleges, with an annual admission capacity of 56,662 students (National Health Profile (NHP) of India, 2021). The Bachelor in Naturopathy and Yogic Sciences (BNYS), which integrates naturopathy, yoga, and modern medical sciences, is a 5.5-year medical degree under India's Ayush system. The program, open to students who have completed 12th grade and have a biology background, is currently offered in 56 state-affiliated medical colleges across India. BNYS graduates are recognized as Class “A” medical practitioners in nearly 20 states contributing to the integrative medicine field (Nair et al., 2023). Naturopathy, recognized by the World Health Organization, follows a principle-based approach to health care, emphasizing “first do no harm”, harnessing the body's natural healing ability, and addressing root causes of illness and patient education (Dunn et al., 2021). In addition to basic medical and life sciences, BNYS students are trained in various complementary and alternative medicine modalities, including lifestyle counseling, mind–body therapies, hydrotherapy, therapeutic fasting, diet therapy, mud therapy, heliotherapy, chromotherapy, magnetotherapy, physiotherapy, ozone therapy, acupressure, and acupuncture (Sadowski et al., 2022; Nair and Nanda, 2014).

Despite students studying traditional systems of medicine facing similar academic stressors as modern medical students, studies on their sleep patterns are limited. While extensive research has been conducted on sleep patterns among modern medical students (Nsengimana et al., 2023), to the best of our knowledge, no studies have specifically examined these factors among students of yoga and naturopathy. This gap is particularly significant, given the fundamental differences in therapeutic approaches between the two medical streams. Unlike modern medical students, who primarily focus on pharmacological interventions, yoga and naturopathy students are trained to emphasize lifestyle modifications as a core component of disease prevention and management. Therefore, understanding their sleep hygiene, sleep quality, and related factors is essential, as it provides insights into whether they integrate these principles into their own lives during medical training. Evaluating their adherence to lifestyle-based practices, including sleep hygiene, could offer a unique perspective on the self-practice of holistic health principles among this cohort. Therefore, this study was conducted to evaluate sleep hygiene, sleep quality, and their correlates among yoga and naturopathic medical students in India.

## Methodology

### Study design and settings

We conducted an online cross-sectional survey among medical students pursuing the BNYS, a 5.5-year undergraduate program in yoga and naturopathy, from 10 medical colleges in 6 states (Madhya Pradesh, Tamil Nadu, Karnataka, Uttar Pradesh, Andhra Pradesh, and Gujarat) of India. The study was approved by an independent ethics committee (IASWAR/P03/2023). All the participants signed a digital informed consent before responding to the survey.

### Sample size and sampling technique

We employed a cluster sampling approach, with each medical college considered a cluster. Cluster sampling was used in this study to enhance feasibility and logistical efficiency while ensuring adequate representation of the target population. Given that students from traditional systems of medicine are dispersed across multiple institutions, selecting entire medical colleges as clusters allowed for a more practical and systematic approach to data collection. The sample size was estimated using OpenEpi at a prevalence of poor sleep quality of 45% (taken from a previous study) (Mishra et al., 2022), absolute precision of 5%, a confidence level of 95%, a design effect of 2 (to account for cluster sampling), and a non-response rate of 50% (considering the high non-response rates for online surveys). The estimated sample size was 1,142.

### Study instrument and data collection

The questionnaire, designed using Google Forms, included a combination of well-established scales, such as the Kuppuswamy Scale, the Pittsburgh Sleep Quality Index (PSQI), the Sleep Hygiene Index (SHI), the Depression Anxiety Stress Scale (DASS-21), and the Internet Addiction Test (IAT). Additionally, the research team incorporated questions related to the demographic characteristics of the study cohort within the same Google Form. This questionnaire was sent to the email addresses of the college heads, who then circulated it to the prospective students (*n* = 2,675), and completed responses were received from 1,151 participants. The coordinators from each medical college facilitated the data collection process, ensuring informed consent before filling out the questionnaire. Data were collected between May and June 2023.

The survey questionnaire encompassed various sociodemographic variables, including age, sex, institution name, years of study, motivations for selecting medical school, average examination grades, social support, and socioeconomic status (assessed using the Kuppuswamy Scale). The Kuppuswamy Scale considers three key factors: the education and occupation of the head of the family and the total monthly family income. This composite scoring system categorizes socioeconomic status into five classes: lower class, upper lower class, lower middle class, upper middle class, and upper class (Wani, 2019).

Lifestyle-related inquiries delved into consumption habits, such as types; the use of substances such as alcohol, tobacco, aerated beverages, and junk foods; and the duration and intensity of physical activity were gathered. The survey also incorporated well-established assessment tools, including the PSQI, the SHI, the DASS-21, and the IAT.

The PSQI is a 19-item self-reported questionnaire consisting of 7 component scores that measure sleep quality, with a global score ranging from 0 to 21. A score >5 indicates poor sleep quality (Buysse et al., 1989). The PSQI has demonstrated acceptable reliability and structural validity (De Moraes et al., 2024).

The SHI is a 13-item questionnaire, where each item was rated on a 5-point scale ranging from 0 (*never*) to 4 (*always*). The total SHI scores vary from 0 to 52, and a higher score indicates poorer sleep hygiene (Mastin et al., 2006). The internal consistency, assessed by Cronbach's alpha, was reported to be 0.64 (Seun-Fadipe et al., 2018).

The psychological health of participants was assessed using the DASS-21 questionnaire, which includes 21 questions organized into 3 subscales with 7 items each, targeting depression, anxiety, and stress. Responses were scored on a scale from 0 (*did not apply to me at all*) to 3 (*applied to me very much*). Total scores for depression, anxiety, and stress were calculated by multiplying the obtained values by 2 (Depression Anxiety Stress Scale-21 (DASS21), n.d.). The tool is reliable and appropriate for assessing symptoms of common mental health, showing sufficient internal consistency with Cronbach's alpha ranging from 0.76 to 0.91 (Le et al., 2017).

We also utilized a 20-item IAT questionnaire to assess characteristics and behaviors associated with compulsive Internet use. Each item is scored using a Likert scale ranging from 0 (*less likely behavior*) to 5 (*most likely behavior*). A total score of 0–30 points indicates a normal level of Internet usage, a score range of 31–49 indicates the presence of a mild level of Internet addiction, scores of 50–79 indicate a moderate level, and scores of 80–100 indicate a severe level of Internet addiction (Ann Liebert and Young, 1998). The IAT is a reliable tool in diagnosing Internet addiction, with Cronbach's alpha coefficient of 0.91 (Moon et al., 2018).

### Statistical analysis

We analyzed the sample of 1,151 participants from 10 different geographical clusters. The data captured in Microsoft Excel^®^ were exported to IBM SPSS version 27 for cleaning and analysis.

During the data cleaning phase, we checked for missing data and data inconsistencies. Sleep hygiene was computed as a composite index of the 13-item sleep hygiene scale, yielding a composite score ranging from 0 to 45. A score < one standard deviation from the mean (score of 0–16) was categorized as good sleep hygiene. A score >1 standard deviation from the mean (score of 30 and above) is considered poor sleep hygiene, and a score of within one standard deviation from the mean (score of 17–29) is considered moderate sleep hygiene. Sleep quality was captured as a composite index based on the PSQI, with a score ranging from 0 to 17. A score of up to 5 was considered good sleep quality, and a score of 6 and above was categorized as poor sleep quality (Park, 2020).

The scores for depression, anxiety, and stress were categorized as normal, mild to moderate, and severe to extremely severe per the DASS 21 severity ratings. The Internet addiction score was categorized as normal (0–30), mild (31–49), and moderate to severe (50 and above) addiction.

We calculated the percentage prevalence and 95% confidence intervals (CIs) to descriptively analyze the study variables. The multinomial logistic regression model was developed with the SHI score as a dependent variable and selected sociodemographic characteristics, depression, anxiety, stress, and Internet addiction as the independent variables. Binary logistic regression analysis was performed to identify the impact of sociodemographic characteristics, DASS-21 variables, and sleep hygiene on sleep quality. Hosmer–Lemeshow's test for goodness of fit indicated that the regression models had an acceptable model fit (*p* > 0.05). The adjusted odds ratios (AORs) and 95% CI were computed. The standard errors of the AOR were adjusted for the cluster effect of the 10 geographical clusters in the sample.

## Results

### Sociodemographic characteristics

The study included 1,151 participants, with a predominant majority being female (79.8%). Nearly half were 1st-year students (49.0%), and most resided in hostels (70.0%). The majority belonged to the lower middle (37.7%) and upper middle (51.5%) socioeconomic classes. Substance use was low, with only 2.5% reporting alcohol use and 0.6% using tobacco. Regarding dietary habits, 56.5% occasionally consumed sweetened beverages, and 54.6% occasionally ate fried/packaged foods. The majority of students (82.6%) engaged in physical activity, but 89.5% used digital devices after 9 p.m. Perceived social support was strong in 44.0% of participants, while 6.9% reported poor support (see Table 1). The average total SHI score of the study sample was 22.49 (±6.34), and the mean PSQI score was 6.52 (±3.01). Sleep duration varied by Internet addiction, depression, anxiety, and stress levels (see the Supplementary material).

**Table 1:** Sociodemographic characteristics of the study sample (*N* = 1,151).

**Variable**	**Males (*n* = 232)**	**Females (*n* = 919)**	**Total (*N* = 1,151)**
Year of study	*n* (%)	*n* (%)	*n* (%)
First year	118 (50.9)	446 (48.5)	564 (49.0)
Second year	31 (13.4)	147 (16.0)	178 (15.5)
Third year	54 (23.3)	172 (18.7)	226 (19.6)
Final year	29 (12.5)	154 (16.8)	183 (15.9)
**Current place of residence**			
Hostel	145 (62.5)	661 (71.9)	806 (70.0)
Rented accommodation	47 (20.3)	96 (10.4)	143 (12.4)
With family	40 (17.2)	162 (17.6)	202 (17.5)
**Socioeconomic status**			
Lower class	18 (7.8)	32 (3.5)	50 (4.3)
Upper lower class	11 (4.7)	29 (3.2)	40 (3.5)
Lower middle class	102 (44.0)	332 (36.1)	434 (37.7)
Upper middle class	95 (40.9)	498 (54.2)	293 (51.5)
Upper class	6 (2.6)	28 (3.0)	34 (3.0)
**Uses alcohol**			
No	218 (94.0)	904 (98.4)	1,122 (97.5)
Yes	14 (6.0)	15 (1.6)	29 (2.5)
**Tobacco use**			
No	230 (99.1)	914 (99.5)	1,144 (99.4)
Yes	2 (0.9)	5 (0.5)	7 (0.6)
**Sweetened sugar beverages**			
Never	64 (27.6)	243 (26.4)	307 (26.7)
Occasionally	100 (43.1)	550 (59.8)	650 (56.5)
Once a week	45 (19.4)	101 (11.0)	146 (12.7)
Daily	23 (9.9)	25 (2.7)	48 (4.2)
**Fried and packaged food consumption**			
Never	35 (15.1)	55 (6.0)	90 (7.8)
Occasionally	98 (42.2)	531 (57.8)	629 (54.6)
Once a week	76 (32.8)	273 (29.7)	349 (30.3)
Daily	23 (9.9)	60 (6.5)	83 (7.2)
**Undertakes mild to vigorous physical activity**			
No	42 (18.1)	98 (10.7)	200 (17.4)
Yes	190 (81.9)	821 (89.3)	951 (82.6)
**Use of mobile/laptop after 9 p.m**.			
No	23 (9.9)	98 (10.7)	121 (10.5)
Yes	209 (90.1)	821 (89.3)	1,030 (89.5)
**Perceived social support from family/friends/teachers/college**			
Strong	110 (47.4)	397 (43.2)	507 (44.0)
Moderate	97 (41.8)	468 (50.9)	565 (49.1)
Poor	25 (10.8)	54 (5.9)	79 (6.9)

### Mental health, Internet addiction, and sleep quality among yoga and naturopathy students

Among the surveyed yoga and naturopathy students (*N* = 1,151), 59.8% [95% CI (56.9, 62.6)] reported poor sleep quality, while 24.7% [95% CI (22.2, 27.2)] had poor sleep hygiene. Nearly 30% of the participants exhibited some degree of Internet addiction, with males showing higher addiction levels [8.2%, 95% CI (5.1, 12.2)]. Anxiety was a significant concern, with 32.8% [95% CI (30.3, 35.6)] experiencing severe to extremely severe symptoms, particularly among females [34.2%, 95% CI (31.1, 37.3)]. Additionally, 42.7% [95% CI (39.8, 45.5)] had mild to moderate depression, and 15.8% [95% CI (13.8, 18.0)] had severe to extremely severe depression. While 67.3% [95% CI (64.6, 70.0)] reported normal stress levels, 8.3% [95% CI (6.8, 10.0)] experienced severe to extremely severe stress (see Table 2).

**Table 2:** Percentage prevalence of depression, anxiety, stress, Internet addiction level, and sleep quality among the yoga and naturopathy students (*N* = 1,151).

**Independent variables**	**Males (*n* = 232)**	**Females (*n* = 919)**	**Total sample (*N* = 1,151)**

	**% [95% CI]**	**% [95% CI]**	**% [95% CI]**
**Depression**			
Normal	45.3 [38.9, 51.7]	40.6 [37.4, 43.8]	41.5 [38.7, 44.4]
Mild to moderate	39.7 [33.5, 46.0]	43.4 [40.2, 46.6]	42.7 [39.8, 45.5]
Severe to extremely severe	15.1 [10.9, 20.1]	16.0 [13.7, 18.5]	15.8 [13.8, 18.0]
**Anxiety**			
Normal	37.5 [31.4, 43.8]	25.7 [22.9, 28.6]	28.1 [25.5, 30.7]
Mild to moderate	34.9 [29.0, 41.2]	40.2 [37.0, 43.3]	39.1 [36.3, 41.9]
Severe to extremely severe	27.6 [22.1, 33.6]	34.2 [31.1, 37.3]	32.8 [30.2, 35.6]
**Stress**			
Normal	72.8 [66.9, 78.3]	65.9 [62.8, 69.0]	67.3 [64.6, 70.0]
Mild to moderate	19.8 [15.0, 25.3]	25.5 [22.7, 28.3]	24.3 [21.9, 26.9]
Severe to extremely severe	7.3 [4.4, 11.2]	8.6 [6.9, 10.5]	8.3 [6.8, 10.0]
**Internet addiction level**			
Normal	61.6 [55.3, 67.7]	72.8 [69.9, 75.6]	70.5 [67.9, 73.1]
Mild	30.2 [24.5, 36.3]	22.1 [19.5, 24.8]	23.7 [21.3, 26.2]
Moderate to severe	8.2 [5.1, 12.2]	5.1 [3.8, 6.7]	5.7 [4.5, 7.2]
**Sleep hygiene**			
Good sleep hygiene	22.4 [17.4, 28.1]	24.2 [21.5, 27.0]	23.8 [21.4, 26.3]
Moderate sleep hygiene	48.7 [42.3, 55.1]	52.2 [49.0, 55.5]	51.5 [48.6, 54.4]
Poor sleep hygiene	28.9 [23.3, 34.9]	23.6 [20.9, 26.4]	24.7 [22.2, 27.2]
**Sleep quality**			
Good	48.7 [42.3, 55.1]	38.1 [35.0, 41.3]	40.2 [37.4, 43.1]
Poor	51.3 [44.9, 57.7]	61.9 [58.7, 65.0]	59.8 [56.9, 62.6]

### Factors associated with sleep hygiene scores among yoga and naturopathy students

The multinomial logistic regression analysis revealed several significant factors associated with sleep hygiene. Among males, engaging in mild physical activity was significantly associated with lower odds of poor sleep hygiene [AOR = 0.20, 95% CI (0.06, 0.67)], and moderate physical activity was similarly associated with reduced odds of poor sleep hygiene [AOR = 0.22, 95% CI (0.06, 0.83)]. Males living with family had significantly lower odds of poor sleep hygiene [AOR = 0.14, 95% CI (0.04, 0.53)], while females residing with family were also significantly less likely to have both poor [AOR = 0.26, 95% CI (0.12, 0.59)] and moderate [AOR = 0.41, 95% CI (0.27, 0.64)] sleep hygiene. Additionally, daily consumption of sweetened sugary beverages was significantly associated with poor sleep hygiene in the total sample [AOR = 2.02, 95% CI (0.66, 6.16)], and moderate to severe Internet addiction was linked to significantly higher odds of poor sleep hygiene in both males [AOR = 21.87, 95% CI (8.96, 53.39)] and females [AOR = 9.57, 95% CI (2.11, 43.11)].

Significant associations were also observed between unhealthy food consumption and sleep hygiene. Consuming fried and packaged foods once a week [AOR = 5.06, 95% CI (2.43, 10.51)] and daily [AOR = 8.52, 95% CI (3.64, 19.98)] were associated with significantly higher odds of poor sleep hygiene in the total sample. The use of mobile/laptop after 9 p.m. was also significantly linked to poor sleep hygiene [AOR = 3.80, 95% CI (1.40, 10.28), for females; AOR = 4.54, 95% CI (0.35, 59.48), for males]. Regarding mental health, individuals with severe to extremely severe depression had significantly higher odds of poor sleep hygiene [AOR = 5.15, 95% CI (1.91, 13.86)], and females with severe to extremely severe anxiety had significantly higher odds of poor sleep hygiene [AOR = 2.31, 95% CI (0.79, 6.77)]. Finally, mild to moderate stress was significantly associated with moderate sleep hygiene in females [AOR = 2.04, 95% CI (1.02, 4.08)]. Socioeconomic status, year of study, and perceived social support were not significantly associated with sleep hygiene outcomes (see Table 3).

**Table 3:** Factors associated with sleep hygiene among BNYS graduates: results of multinomial logistic regression.

**Independent variables**	**Male (*****n*** = **232)**		**Female (*****n*** = **919)**		**Total sample (*****N*** = **1,151)**	
	**Moderate sleep hygiene**	**Poor sleep hygiene**	**Moderate sleep hygiene**	**Poor sleep hygiene**	**Moderate sleep hygiene**	**Poor sleep hygiene**
**Sex of the respondent**	**AOR [95% CI]**	**AOR [95% CI]**	**AOR [95% CI]**	**AOR [95% CI]**	**AOR [95% CI]**	**AOR [95% CI]**
**Male subjects (ref)**						
Female subjects	NA	NA	NA	NA	0.98 [0.60, 1.61]	0.65 [0.31, 1.39]
**Year of study**						
**Fourth year (ref)**						
First year	0.78 [0.21, 2.87]	0.67 [0.18, 2.43]	0.87 [0.53, 1.41]	0.59 [0.24, 1.44]	0.85 [0.59, 1.22]	0.66 [0.34, 1.26]
Second year	0.56 [0.13, 2.41]	0.16 [0.01, 1.44]	1.48 [0.64, 3.45]	1.13 [0.36, 3.52]	1.19 [0.58, 2.48]	0.90 [0.33, 2.51]
Third year	0.68 [0.11, 4.08]	0.45 [0.06, 3.17]	1.36 [0.68, 2.75]	1.16 [0.27, 4.95]	1.13 [0.63, 2.02]	0.95 [0.34, 2.64]
**Physical activity**						
**Vigorous (ref)**						
Mild	0.35 [0.12, 1.00]	0.20 [0.06, 0.67]^*^	1.13 [0.15, 8.54]	NA	0.72 [0.20, 2.57]	0.64 [0.14, 2.86]
Moderate	0.38 [0.12, 1.15]	0.22 [0.06, 0.83]^*^	1.01 [0.15, 6.75]	NA	0.67 [0.21, 2.19]	0.81 [0.21, 3.16]
**Socioeconomic status**						
**Lower (ref)**						
Lower upper	NA	NA	1.18 [0.20, 6.92]	0.65 [0.04, 9.59]	0.89 [0.24, 3.28]	0.67 [0.07, 6.39]
Lower middle			1.49 [0.24, 9.40]	1.19 [0.20, 7.33]	0.90 [0.22, 3.70]	1.05 [0.31, 3.54]
Upper middle	NA	NA	1.80 [0.30, 10.96]	1.09 [0.21, 5.75]	0.98 [0.25, 3.87]	1.08 [0.32, 3.62]
Upper			1.44 [0.14, 14.50]	1.03 [0.07, 16.48]	0.61 [0.10, 3.91]	0.93 [0.10, 8.90]
**Current place of residence**						
**Hostel (ref)**						
Outside residence	1.16 [0.34, 3.96]	1.18 [0.50, 2.76]	0.95 [0.62, 1.46]	0.99 [0.44, 2.20]	1.09 [0.74, 1.61]	1.08 [0.60, 1.94]
Family	0.56 [0.31, 1.09]	0.14 [0.04, 0.53]^**^	0.41 [0.27, 0.64]^**^	0.26 [0.12, 0.59]^**^	0.43 [0.30, 0.63]^**^	0.23 [0.11, 0.48]^**^
**Sweetened sugary beverages**						
**Never (ref)**						
Occasionally	2.47 [0.90, 3.96]	2.45 [0.63, 9.62]	1.18 [0.85, 1.64]	1.30 [0.63, 2.70]	1.42 [0.93, 2.16]	1.58 [0.75, 3.33]
Once a week	1.92 [0.31, 11.97]	1.45 [0.21, 9.89]	0.66 [0.37, 1.17]	0.86 [0.29, 2.54]	0.93 [0.69, 1.25]	1.07 [0.48, 2.35]
Daily	4.11 [0.28, 59.47]	2.41 [0.06, 96.06]	0.86 [0.17, 4.30]	1.79 [0.21, 15.47]	1.54 [0.81, 2.90]	2.02 [0.66, 6.16]
**Fried and packaged food consumption**						
**Never (ref)**						
Occasionally	2.05 [0.99, 4.24]	0.39 [0.08, 2.02]	3.31 [1.63, 6.75]^**^	5.62 [0.61, 51.48]	2.64 [1.59, 4.40]^**^	1.53 [0.45, 5.15]
Once a week	2.93 [0.76, 11.24]	1.41 [0.31, 6.40]	5.06 [2.43, 10.51]^**^	9.44 [0.86, 103.58]	4.03 [2.18, 7.45]^**^	2.89 [0.74, 11.34]
Daily	2.49 [0.13, 44.95]	2.31 [0.09, 57.65]	8.52 [3.64, 19.98]^**^	22.87 [2.98, 175.39]^**^	5.53 [2.43, 12.57]^**^	6.04 [1.79, 20.44]^**^
**Use of mobile/laptop after 9 p.m**.						
**No (ref)**						
Yes	1.80 [0.67, 4.85]	4.54 [0.35, 59.48]	2.37 [1.19, 4.73]^*^	3.80 [1.40, 10.28]^**^	2.33 [1.30, 4.20]^**^	3.69 [1.40, 9.72]
**Perceived social support from family/friends/teachers/college**						
**Poor (ref)**						
Moderate	4.63 [1.04, 20.48]^*^	1.91 [0.33, 11.19]	0.62 [0.17, 2.27]	0.38 [0.10, 1.48]	1.39 [0.73, 2.63]	0.78 [0.29, 2.10]
Strong	1.72 [0.88, 3.38]	0.44 [0.21, 0.93]^*^	0.54 [0.18, 1.64]	0.28 [0.06, 1.25]	0.69 [0.21, 2.24]	0.47 [0.20, 1.15]
**Sex of the respondent**	**AOR [95% CI]**	**AOR [95% CI]**	**AOR [95% CI]**	**AOR [95% CI]**	**AOR [95% CI]**	**AOR [95% CI]**
**Internet addiction**						
**Normal (ref)**						
Mild	3.93 [2.07, 7.48]^**^	756 [2.36, 24.21]^**^	1.36 [0.75, 2.47]	1.79 [0.78, 4.13]	1.55 [1.02, 2.35]^*^	2.23 [1.21, 4.12]^*^
Moderate to severe	10.42 [3.46, 31.44]^**^	21.87 [8.96, 53.39]^**^	3.98 [0.27, 58.85]	8.48 [0.48, 148.40]	4.31 [1.45, 16.24]^*^	9.57 [2.11, 43.11]^**^
**Depression**						
**Normal (ref)**						
Mild to moderate	1.99 [0.23, 16.53]	5.72 [0.62, 52.28]	1.96 [1.00, 3.84]	2.70 [0.67, 10.84]	1.87 [0.96, 3.65]	3.01 [1.01, 9.00]^*^
Severe to extremely severe	0.54 [0.05, 5.84]	5.62 [0.11, 281.05]	2.36 [1.29, 4.31]^**^	4.54 [1.56, 13.03]^**^	1.86 [0.96, 3.61]	5.15 [1.91, 13.86]^**^
**Anxiety**						
**Normal (ref)**						
Mild to moderate	2.88 [0.46, 18.05]	1.00 [0.19, 5.13]	1.96 [1.26, 3.04]^**^	1.95 [1.03, 3.70]^*^	2.19 [1.57, 3.06]^**^	1.63 [0.97, 2.71]
Severe to extremely severe	5.07 [1.05, 24.32]^*^	1.82 [0.33, 10.10]	1.69 [0.85, 3.39]	2.31 [0.79, 6.77]	0.69 [0.21, 2.24]^**^	1.83 [0.96, 3.49]
**Stress**						
**Normal (ref)**						
Mild to moderate	0.40 [0.12, 1.34]	0.49 [0.11, 2.26]	2.04 [1.02, 4.08]^*^	2.73 [0.90, 8.25]	1.39 [0.73, 2.63]	1.84 [0.82, 4.15]
Severe to extremely severe	0.37 [0.02, 6.63]	0.52 [0.02, 16.40]	0.80 [0.20, 3.09]	1.26 [0.40, 3.93]	0.69 [0.21, 2.24]	1.12 [0.40, 3.13]

Dependent variable: sleep hygiene scores, good sleep hygiene (ref), poor sleep hygiene; and moderate sleep hygiene. For the variables physical activity and socioeconomic status the frequencies of the independent variables when stratified by sex are very minimal and generated non-comprehensible odds ratios. The standard errors of the adjusted odds ratios are adjusted for the 10 geographical clusters in the sample.

AOR, adjusted odds ratio; CI, confidence interval; ref, reference category; NA, not applicable in the model as the model was exclusively for males/females.

^*^p < 0.05.

^**^p < 0.01.

### Factors associated with sleep quality among yoga and naturopathy students

Binary logistic regression analysis for a sample of 1,151 respondents revealed several significant factors associated with good sleep quality. For females, being in the 3rd year of study was significantly linked to better sleep quality (AOR = 1.82, *p* = 0.006), while no significant relationship was found for males. In the total sample, 3rd-year students showed better sleep quality (AOR = 1.50, *p* = 0.025). Socioeconomic status also played a significant role, with females from the lower middle socioeconomic group more likely to have good sleep quality (AOR = 1.83, *p* = 0.001), and a similar trend was observed in the total sample (AOR = 1.88, *p* = 0.018). Additionally, place of residence was an important factor, with males living with family showing significantly better sleep quality (AOR = 5.94, *p* < 0.001), and this association remained significant in the total sample (AOR = 1.46, *p* = 0.025).

Lifestyle factors, such as sweetened sugary beverage consumption and fried food intake, were associated with sleep quality. For males, daily consumption of sweetened sugary beverages was negatively associated with good sleep quality (AOR = 0.39, *p* < 0.001), while in the total sample, daily consumption of fried and packaged foods was linked to poorer sleep quality (AOR = 1.74, *p* = 0.020). The use of mobile/laptop after 9 p.m. was significantly associated with poor sleep quality, particularly for males (AOR = 0.24, *p* = 0.030) and in the total sample (AOR = 0.74, *p* = 0.043). Furthermore, females with strong perceived social support were more likely to have good sleep quality (AOR = 1.96, *p* = 0.041). Psychological factors, particularly severe to extremely severe stress, were significantly associated with poor sleep quality in the total sample (AOR = 0.55, *p* = 0.014), and mild to moderate depression showed a significant association with sleep quality in males (AOR = 0.94, *p* = 0.049). Additionally, in the total sample, moderate sleep hygiene was significantly associated with good sleep quality (AOR = 0.68, *p* < 0.001; see Table 4).

**Table 4:** Factors associated with good sleep quality: results of binary logistic regression (*N* = 1,151).

**Independent variables**	**Male subjects (*****n*** = **232)**		**Female subjects (*****n*** = **919)**		**Total sample (*****N*** = **1,151)**	
	**AOR [95% CI]**	***p*** **-value**	**AOR [95% CI]**	***p*** **-value**	**AOR [95% CI]**	***p*** **-value**
**Sex of the respondent**						
**Male subjects (ref)**						
Female subjects	NA	NA	NA	NA	0.76 [0.51, 1.39]	0.368
**Year of study**						
**Fourth year (ref)**						
First year	0.99 [0.45, 2.19]	0.985	1.38 [0.93, 2.05]	0.108	1.25 [0.87, 1.81]	0.226
Second year	3.63 [1.98, 6.66]	< 0.001	0.87 [0.41, 1.81]	0.702	0.99 [0.50, 1.99]	0.983
Third year	0.98 [0.39, 2.48]	0.972	1.82 [1.19, 2.78]	0.006	1.50 [1.05, 2.13]	0.025
**Physical activity**						
**Vigorous (ref)**						
Mild	1.03 [0.35, 3.02]	0.964	0.58 [0.17, 1.95]	0.380	0.70 [0.32, 1.53]	0.374
Moderate	1.25 [0.65, 2.40]	0.501	0.61 [0.21, 1.81]	0.370	0.75 [0.43, 1.30]	0.300
**Socioeconomic status**						
**Lower (ref)**						
Lower upper	0.30 [0.02, 4.23]	0.375	2.39 [1.27, 4.49]	0.007	1.76 [0.79, 3.91]	0.169
Lower middle	1.12 [0.31, 4.00]	0.866	1.83 [1.28, 2.60]	0.001	1.88 [1.11, 3.17]	0.018
Upper middle	1.47 [0.44, 4.97]	0.534	1.35 [0.91, 1.99]	0.134	1.53 [0.98, 2.40]	0.062
Upper	0.22 [0.06, 0.84]	0.026	1.47 [0.89, 2.42]	0.133	1.36 [0.82, 2.27]	0.238
**Current place of residence**						
**Hostel (ref)**						
Outside residence	1.64 [0.84, 3.20]	0.148	1.31 [0.79, 2.19]	0.288	1.33 [0.87, 2.04]	0.188
Family	5.94 [2.91, 12.13]	< 0.001	1.17 [0.87, 1.58]	0.299	1.46 [1.05, 2.04]	0.025
**Sweetened sugary beverages**						
**Never (ref)**						
Occasionally	0.80 [0.27, 2.34]	0.681	1.14 [0.68, 1.93]	0.614	1.07 [0.74, 1.54]	0.722
Once a week	1.69 [1.06, 2.69]	0.029	1.53 [0.96, 2.43]	0.075	1.48 [0.97, 2.26]	0.069
Daily	0.39 [0.29, 0.53]	< 0.001	0.85 [0.28, 2.56]	0.774	0.69 [2.91, 1.64]	0.403
**Fried and packaged food consumption**						
**Never (ref)**						
Occasionally	0.84 [0.53, 1.33]	0.454	0.94 [0.39, 2.29]	0.892	0.91 [0.59, 1.43]	0.693
Once a week	1.02 [0.47, 2.23]	0.952	0.77 [0.28, 2.12]	0.616	0.84 [0.50, 1.43]	0.529
Daily	2.04 [0.76, 5.45]	0.154	1.61 [0.84, 3.07]	0.150	1.74 [1.09, 2.78]	0.020
**Use of mobile/laptop after 9 p.m**.						
**No (ref)**						
Yes	0.24 [0.07, 0.87]	0.030	0.82 [0.50, 1.35]	0.436	0.74 [0.55, 0.99]	0.043
**Perceived social support from family/friends/teachers/college**						
**Poor (ref)**						
Moderate	0.75 [0.22, 2.49]	0.635	1.67 [0.91, 3.07]	0.095	1.15 [0.72, 1.83]	0.549
Strong	1.11 [0.38, 3.28]	0.842	1.96 [1.03, 3.74]	0.041	1.44 [0.88, 2.37]	0.144
**Internet addiction**						
**Normal (ref)**						
Mild	0.91 [0.45, 1.83]	0.793	1.09 [0.65, 1.82]	0.745	1.07 [0.67, 1.72]	0.757
Moderate to severe	0.42 [0.14, 1.28]	0.126	1.06 [0.53, 2.12]	0.876	0.88 [0.52, 1.50]	0.641
**Depression**						
**Normal (ref)**						
Mild to moderate	0.46 [0.21, 0.99]	0.049	0.98 [0.65, 1.46]	0.904	0.94 [0.62, 1.42]	0.762
Severe to extremely severe	0.61 [0.15, 2.45]	0.485	0.96 [0.46, 1.99]	0.906	0.89 [0.48, 1.65]	0.718
**Anxiety**						
**Normal (ref)**						
Mild to moderate	1.31 [0.68, 2.54]	0.416	0.76 [0.49, 1.16]	0.197	0.79 [0.57, 1.09]	0.147
Severe to extremely severe	0.61 [0.15, 2.45]	0.583	0.50 [0.27, 0.93]	0.027	0.57 [0.30, 1.08]	0.084
**Stress**						
**Normal (ref)**						
Mild to moderate	0.46 [0.18, 1.16]	0.099	0.77 [0.55, 1.10]	0.151	0.72 [0.49, 1.07]	0.108
Severe to extremely severe	0.51 [0.07, 3.43]	0.486	0.58 [0.38, 0.87]	0.008	0.55 [0.35, 0.89]	0.014
**Sleep hygiene**						
**Good (ref)**						
Moderate	0.69 [0.28, 1.66]	0.406	0.69 [0.52, 0.93]	0.014	0.68 [0.55, 0.84]	< 0.001
Poor	1.64 [0.55, 4.87]	0.373	0.48 [0.28, 0.83]	0.008	0.62 [0.36, 1.07]	0.087

Dependent variable: sleep quality: Good (ref), Poor. Sleep quality was captured based on the Pittsburgh Sleep Quality Scale. A sleep quality score of 0–5 was considered good, and a sleep quality score of 6 and above was considered poor. The standard errors of the adjusted odds ratios are adjusted for the 10 geographical clusters in the sample.

AOR, adjusted odds ratio; ref, reference category; Ref, reference category; NA, not applicable for inclusion in the model as the model was exclusively among males and females.

## Discussion

The present study investigated sleep hygiene, sleep quality, and the specific factors influencing them among yoga and naturopathy medical students from 10 different clusters in India. A sample of 1,151 1st- to 4th-year yoga and naturopathy students were included in this study. In our study, 59.8% of the study sample reported poor sleep quality. This finding aligns with a recent study conducted on 284 undergraduate medical students in which the prevalence of poor sleep quality was reported at 45% (Mishra et al., 2022). In addition, a systematic review and meta-analysis examining sleep quality among medical students reported a prevalence rate of 55.64% for poor sleep quality (Wani, 2019). This finding indicates that, much like other medical students, inadequate sleep quality is prevalent among yoga and naturopathy medical students, thus requiring attention.

Nearly 51.5% of our participants reported moderate sleep hygiene scores while 24.7% reported poor sleep hygiene scores. The prevalence is comparatively lower than findings from previous studies conducted on medical students in various countries: India (72.9%), Qatar (70%), Iran (57.5%), and Ethiopia (48.1%) (Molla and Wondie, 2021; Arora et al., 2015; Ali et al., 2023; Yazdi et al., 2016). This difference in sleep hygiene could be attributed to the unique academic curriculum followed by yoga and naturopathic students. Unlike conventional medical programs, yoga and naturopathic education integrates holistic approaches, including yoga, lifestyle education, and a focus on overall wellbeing. These elements encourage practices that promote better sleep hygiene, such as stress management, mindfulness, and physical health. Yoga and naturopathic programs' emphasis on a balanced lifestyle likely contributes to the healthier sleep hygiene behaviors observed in our participants. However, despite the lower prevalence, poor sleep hygiene is still present among yoga and naturopathy students, which warrants attention.

The high prevalence of anxiety and depression among students is a significant concern, with 32.8% experiencing severe to extremely severe anxiety and 15.8% reporting severe to extremely severe depression. Our findings highlight a strong association between mental health challenges and poor sleep quality. Specifically, individuals with severe to extremely severe depression had significantly higher odds of poor sleep hygiene scores (AOR = 5.15), and females with severe to extremely severe anxiety had significantly higher odds of poor sleep hygiene (AOR = 2.31). Additionally, mild to moderate stress was significantly associated with moderate sleep hygiene scores in females (AOR = 2.04). Regarding sleep quality, severe to extremely severe stress was significantly associated with poor sleep quality in the total sample (AOR = 0.55). In addition, in our study, females with strong perceived social support were more likely to have good sleep quality (AOR = 1.96).

The literature suggests a bidirectional relationship between sleep quality and mental health (Fang et al., 2019). Depression is significantly linked to poor sleep quality, as reported by earlier studies (Garmabi et al., 2024; Joo et al., 2022). Another study reported that anxiety and/or escalating levels of anxiety negatively affect sleep quality (Manzar et al., 2021). Furthermore, stress can result in sleep fragmentation, reduced slow-wave sleep, and poor sleep efficiency (Kim and Dimsdale, 2007). In this study, females, especially those experiencing severe anxiety and stress, were more likely to report poor sleep hygiene scores than their male counterparts. This observation aligns with existing research indicating that women are more susceptible to mental health issues, such as anxiety and depression, which, in turn, can disrupt sleep (Strand et al., 2021; Fatima et al., 2016). Factors such as hormonal fluctuations, caregiving responsibilities, and societal pressures may exacerbate stress and anxiety in women, contributing to poorer sleep outcomes (Fatima et al., 2016; Baker et al., 2018). This highlights that poor mental health is not exclusive to conventional medical students but is also common among students of traditional medicine, including yoga and naturopathy. The findings underscore the need for targeted mental health support and sleep hygiene interventions within yoga and naturopathy programs to improve sleep quality. Incorporating practical coping strategies and offering additional training on sleep hygiene, especially for female students, may further enhance their sleep quality and overall wellbeing.

Our findings indicate that students residing with their families had significantly lower odds of poor sleep hygiene compared to those living in hostels. The home environment, offering emotional support, a stable routine, and greater comfort may contribute to better sleep quality. In contrast, hostel living, often linked to higher stress and noise, could negatively impact sleep hygiene. Earlier studies among medical undergraduates also substantiate our findings, which reported better sleep quality among home dwellers compared to hostellers (Tripathi, 2020; Aftab Khan and Rehana Malik, 2021). The results highlight the importance of considering living arrangements when addressing sleep quality, suggesting that fostering supportive home environments within the hostels may improve students' sleep outcomes.

In the total sample, consuming fried and packaged foods once a week (AOR = 5.06) and daily (AOR = 8.52) were significantly linked to higher odds of poor sleep hygiene. In addition, daily consumption of fried and packaged foods was associated with poorer sleep quality (AOR = 1.74). This underscores the intricate relationship between diet and sleep quality. A diet rich in tryptophan, isoflavones, selenium, vitamin C, vitamin D, and fiber has been recognized for its role in regulating the sleep–wake cycle (Sousa et al., 2020). On the contrary, consumption of poor-quality, packed, and processed foods is linked to elevated levels of inflammatory markers such as C-reactive protein and interleukin-6, contributing to sleep disturbances (Sousa et al., 2020). This highlights the need to advocate for healthy eating habits as a strategic approach to enhancing sleep hygiene and quality among medical students.

Our results suggest that using mobile phones and laptops after 9 p.m. was significantly associated with both poor sleep hygiene and sleep quality. In the total sample, individuals who used their mobile or laptop after 9 p.m. had significantly higher odds of poor sleep hygiene (AOR = 3.80 for females, AOR = 4.54 for males) and poorer sleep quality (AOR = 0.74, *p* = 0.043). Moreover, the presence of moderate to severe Internet addiction was linked to significantly higher odds of poor sleep hygiene in both males (AOR = 21.87) and females (AOR = 9.57). Existing evidence supports the notion that screen usage is associated with poor sleep quality and insomnia, with the blue light emitted from screens suppressing melatonin production, leading to circadian disruption and sleep impairment (Sinha et al., 2022; Adams et al., 2013). In addition, Internet addiction showed a dose–response relationship, with moderate to severe addiction strongly associated with both poor sleep hygiene and sleep quality. These results are consistent with studies by Tokiya et al. (2020) and Tahir et al. (2021). With increasing trends of using digital media for both academic and non-academic purposes, the findings from this study, that increased screen time, Internet addiction, and subsequent sleep quality deterioration warrants serious attention. Such disruptions could be attributed to the increased workload from assignments or excessive engagement with social media, both of which may interfere with adherence to good sleep hygiene practices. Despite yoga and naturopathy students' awareness of the importance of sleep hygiene, high screen time remains prevalent, highlighting the need for strategies such as sleep hygiene education programs to address this behavior. However, further studies are needed to explore the underlying mechanisms and identify effective interventions.

Our analysis showed that both mild and moderate physical activity were significantly linked to lower odds of poor sleep hygiene in males, while no such relationship was observed in females. Physical activity is known to support better sleep by enhancing melatonin production a key hormone in regulating sleep–wake cycles improving sleep quality (Korkutata et al., 2025). The lack of a similar association in females is noteworthy and may point to sex-specific differences in how exercise influences sleep. Studies suggest that women often experience distinct sleep patterns, hormonal variations, and psychosocial factors that may alter the effects of physical activity on sleep. In addition, the higher levels of stress, anxiety, and depression more commonly reported among females (Strand et al., 2021) might offset the sleep benefits typically associated with exercise. These findings underscore the need for further research to better understand how sex-related factors interact with physical activity to shape sleep hygiene outcomes.

Our analysis showed that being in the 3rd year of study was significantly associated with better sleep quality, with 3rd-year students demonstrating improved sleep outcomes overall (AOR = 1.50). This association may reflect greater adaptation to academic demands and more effective time management skills developed as students advance in their studies. As they progress, students might establish more structured routines and coping strategies, positively influencing their sleep patterns. Socioeconomic status also emerged as a significant factor in sleep quality. Students from the lower middle socioeconomic group were more likely to report good sleep quality (AOR = 1.88). Interestingly, when analyzed by sex, these associations were significant primarily among females. This suggests that sex-specific factors may interact with both academic progression and socioeconomic status to shape sleep outcomes. Further research is needed to unravel these underlying mechanisms.

Nevertheless, these findings highlight the need for prioritizing mental health interventions among yoga and naturopathy students, particularly addressing anxiety, depression, screen time, physical inactivity, unhealthy eating habits, and Internet addiction, to improve sleep quality. Promoting healthy sleep practices, such as limiting late-night screen use, encouraging balanced diets, and managing mental health symptoms, could play a crucial role in enhancing sleep outcomes. In addition, fostering social support systems, including counseling and academic support groups, may help students better navigate academic and social pressures that adversely affect their sleep.

### Strengths and limitations

To our knowledge, this study is the first to report on sleep hygiene and associated sleep quality among medical students from traditional medical systems. The large sample size, representation from diverse socio-geographical locations, and using a standardized questionnaire to assess sleep quality, sleep hygiene, and Internet addiction are key strengths of this study. Moreover, using AORs provided a more accurate understanding of the relationships between variables while controlling for potential confounders. In addition, the study's focus on a specific student population, that is, yoga and naturopathy students, offers valuable insights that could inform tailored interventions. However, the cross-sectional design of this study is a limitation. We could not determine whether mood disorder symptoms predated enrolment or emerged during the course, limiting our ability to assess temporal associations. In addition, we collected data on overall screen exposure without distinguishing between academic and entertainment use, preventing us from assessing their specific impacts on sleep hygiene. Furthermore, the majority of our participants were 1st-year BNYS students, predominantly female, and from an upper middle–class background. This may limit the generalizability of our findings to the broader population of yoga and naturopathy students.

## Conclusion

Although less common compared to modern medical students, poor sleep hygiene and sleep quality are still prevalent among yoga and naturopathy medical students, despite their early exposure to lifestyle principles compared to their medical counterparts. Poor sleep hygiene, anxiety, stress, poor dietary preferences, and Internet addiction were identified as the factors that worsen sleep quality. This indicates the need for policies to educate students about the importance of sleep hygiene and its subsequent impact on sleep quality and health.

## Data Availability

The raw data supporting the conclusions of this article will be made available by the authors, without undue reservation.
