# Supplementary material for: Sleep hygiene and sleep quality among yoga and naturopathy medical students in India: a multisite cross-sectional study
Source: Front Sleep. 2025 Apr 9;4:1459750. doi: 10.3389/frsle.2025.1459750 (PMC12713989; doi:10.3389/frsle.2025.1459750)

Supplementary Figure: Sex-Stratified Variation in Sleep Duration Based on Internet Addiction, Depression, Anxiety, and Stress Levels


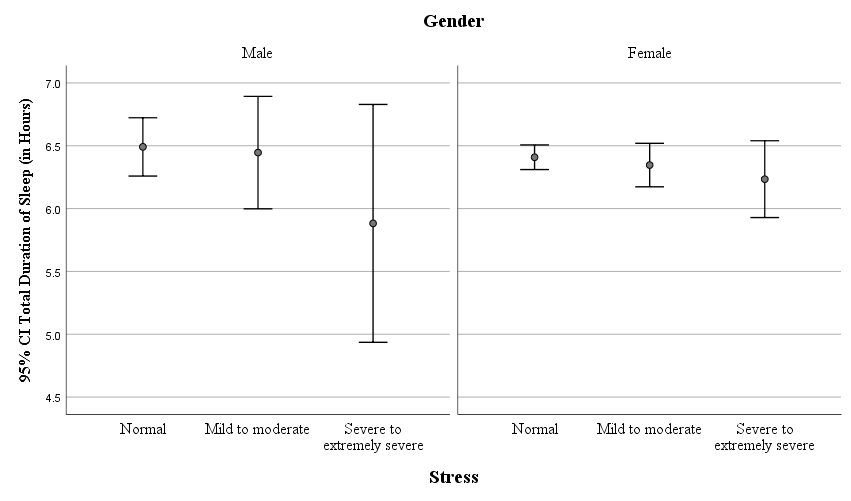


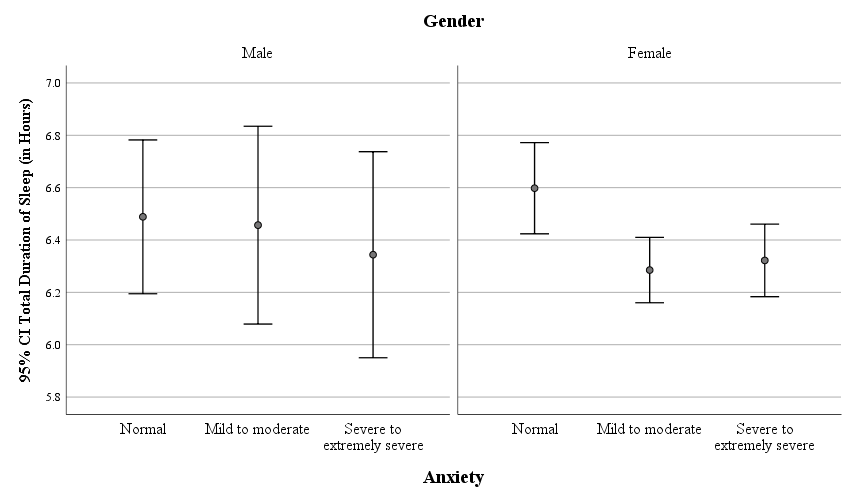


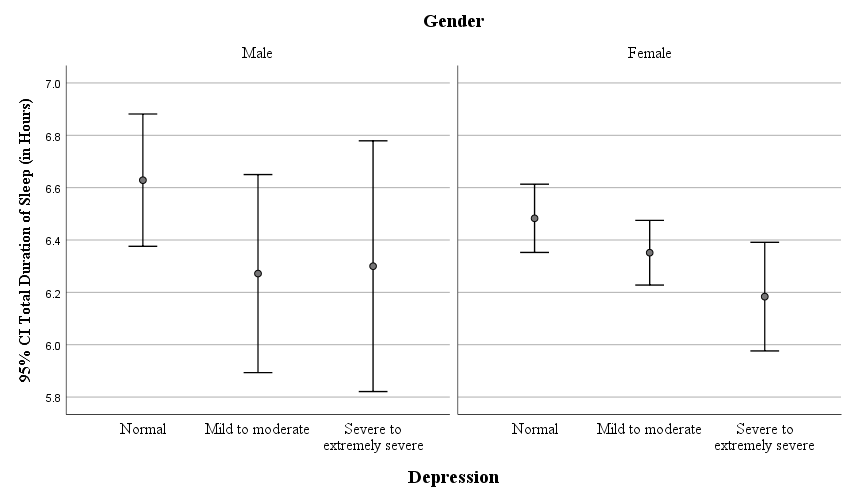


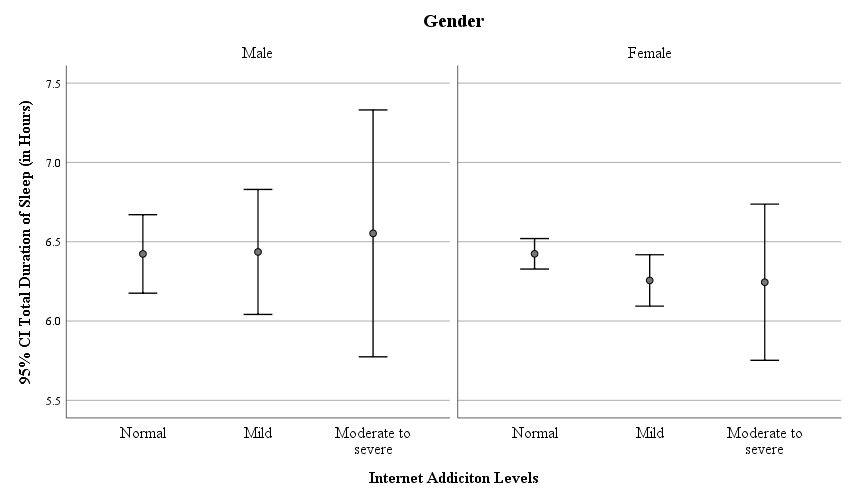

Supplement: Supplementary file 1 [file Table_1.docx]
